# Working memory load impairs transfer learning in human adults

**DOI:** 10.1007/s00426-023-01795-y

**Published:** 2023-01-27

**Authors:** Leonie J. T. Balter, Jane E. Raymond

**Affiliations:** 1 Department of Clinical Neuroscience, Karolinska Institutet, 171 77 Stockholm, Sweden; 2 Stress Research Institute, Department of Psychology, Stockholm University, 114 19 Stockholm, Sweden; 3 School of Psychology, University of Birmingham, Birmingham, B15 2TT UK

## Abstract

Transfer of learning refers to successful application of previously acquired knowledge or skills to novel settings. Although working memory (WM) is thought to play a role in transfer learning, direct evidence of the effect of limitations in WM on transfer learning is lacking. To investigate, we used an acquired equivalence paradigm that included tests of association and transfer learning. The effects of imposing an acute WM limitation on young adults was tested (within-subjects design: *N* = 27 adults; *M*_*age*_ = 24 years) by conducting learning transfer tests concurrent with a secondary task that required carrying a spatial WM load when performing the learned/transfer trial (Load condition) to acutely limit WM resources or no WM load (No-Load condition; WM was unloaded prior to performing the learned/transfer trial). Analyses showed that although success on the transfer trials was high in the No-Load condition, performance dropped to chance in the Load condition. Performance on tests of learned associations remained high in both conditions. These results indicate that transfer of learning depends on access to WM resources and suggest that even healthy young individuals may be affected in their ability to cross-utilize knowledge when cognitive resources become scarce, such as when engaging in two tasks simultaneously (e.g., using satellite navigation while driving).

**Supplementary Information:**

The online version contains supplementary material available at 10.1007/s00426-023-01795-y.

## Introduction

We do not learn everything from scratch when we attempt to learn new things. Instead, we transfer and leverage our knowledge from what we have learnt in the past. Such transfer of learning—the application of learned behavior to novel settings—is an important cognitive skill that allows continual adaptation to new environments, technologies, and people. For example, when using public transport in a novel city, skills and knowledge learned from a familiar transport system are applied and generalized to the novel environment. Of the core cognitive functions underpinning transfer of learning, some data suggest that working memory (WM) mechanisms may play an important role.

Transfer of learning is typically assessed using an acquired equivalence paradigm (Myers et al., 2003). This involves an initial training phase to establish generalization (equivalence) between two independent stimuli. A Transfer Testing phase is used to demonstrate learning of that generalization (transfer) and to test the learned associations. The latter involves accessing long-term memory (LTM) without necessarily using WM resources and likely depends on dopaminergic activity in the basal ganglia (Shohamy et al., 2008). Performance on tests of transfer learning appear to depend heavily on hippocampal function (Myers et al., 2003, 2008), possibly implicating processes that involve spatial WM. Evidence for this view comes from numerous neuropsychological studies linking hippocampal damage to deficits in transfer learning (see Moustafa et al., 2010 for a review) and a large literature indicating that spatial WM is dependent on hippocampal function (Piekema et al., 2006). In addition, in a sample of older adults, spatial WM positively correlated with transfer learning accuracy (*r* = 0.571), although this effect was not observed in the younger adults. Those older adults with poorer WM were also worse at acquiring associations (*r* = 0.500) (Weiler et al., 2008). Thus, although direct evidence is lacking, WM is a tentative candidate cognitive mechanism to underpin successful transfer of learning. If so, then circumstances that limit WM capacity should be associated with reduced capacity for transfer learning, a possibility we tested here.

Numerous prior studies on healthy young adults have shown that WM capacity available for one task can be acutely reduced by engaging in a second, concurrent WM task (De Fockert et al., 2001; Lavie & De Fockert, 2005; Yoo et al., 2004). Here, we exploited this finding to examine the role of WM in transfer learning. We conducted an experiment on young adults using an acquired equivalence paradigm that tested transfer learning under conditions that required them to carry a concurrent WM load, thereby reducing available WM capacity for transfer learning, or no WM load (i.e., unloading WM prior to performing the learned/transfer trial). Loss in transfer performance in the Load condition would indicate that WM is necessary to transfer prior learning to novel situations.

## Methods

### Participants

Twenty-seven young healthy adults (16 females) (*M* = 24 years of age, SD = 5, range 18–40) were recruited from the University of Birmingham and via public (online) advertisements. All completed health and demographics questionnaires prior to participation. Individuals who reported a history of neurological, psychiatric or inflammatory disorders (e.g., rheumatoid arthritis, inflammatory bowel disease) or use of anti-depressant, anti-histamine, or anti-inflammatory medication during the past 7 days were excluded. Participants reported normal or corrected-to-normal vision and participated for course credit or money, after giving informed consent. To maximize performance, performance-based monetary compensation was provided (maximum of £3 per session). All procedures were approved by the University of Birmingham Research Ethics Committee.

### General procedures

A within-subjects design was used such that participants performed the Acquired Equivalence Task twice using an animal or fruit version of the task on different days, scheduled at least 1 day apart. During the first visit, a spatial working memory task was completed followed by the Acquired Equivalence Task. Half of the participants were assigned to the Load condition in the first session and half to the No-Load condition. In the second session, only the Acquired Equivalence Task was completed. The alternate WM condition and alternate task versions were used in the second session, such that WM condition order and task version were fully crossed.

### Sample size

The *Superpower* package in R (RStudio, Inc., Boston, MA URL) was used to approximate the required sample size. Using a power of 80% and an alpha of 0.05 suggests that 15 participants per condition is adequate to detect large effect sizes (i.e., ~ 25% of variance explained) for within-within interactions. The effect of a concurrent WM load on transfer learning performance has not been assessed before, therefore the effect size estimation was based on Weiler et al., 2008, reporting a correlation of *r* = 0.571 between visuospatial working memory and transfer performance. To account for attrition, failure to reach the learning criteria in the training phases, and to accommodate the possibility of medium effect sizes (based on unpublished data from our group finding impaired transfer learning in older versus young adults, ~ 17% of variance explained), we planned to recruit 25 participants.

### Apparatus

A computer (Core i7) running PsychoPy v 1.78.01 (Peirce, 2007) recorded data via a keyboard and presented visual stimuli on a 68-cm ASUS monitor (60-Hz refresh rate, 1280 X 1024 resolution) viewed from approximately 60 cm.

## Measures

### Spatial working memory

The spatial working memory (WM) test consisted of two parts. Part one assessed forward spatial WM capacity and part two backward spatial WM capacity. Each trial started with nine white boxes presented at random locations on a gray screen. One of the boxes turned blue for 1000 ms upon which another box turned blue. The participant was asked to click the boxes that changed color in the same order as presented. Three trials of each sequence were completed. Progressively more boxes changed color after two out of three trials of each sequence were correctly tapped. If two or more errors at the same sequence length were made, the first part was terminated and participants were prompted with the instruction of the second part. Part two was similar to part one except that the participant was asked to click the boxes that changed color in the reverse order as presented. If two or more errors at the same sequence length were made, the spatial WM test was terminated. Outcome measures were maximum forward spatial WM span and maximum backward spatial WM span. The maximum possible score was nine. The spatial WM test was completed during the first session.

### Acquired Equivalence Task

#### Stimuli

**Fig. 1:**
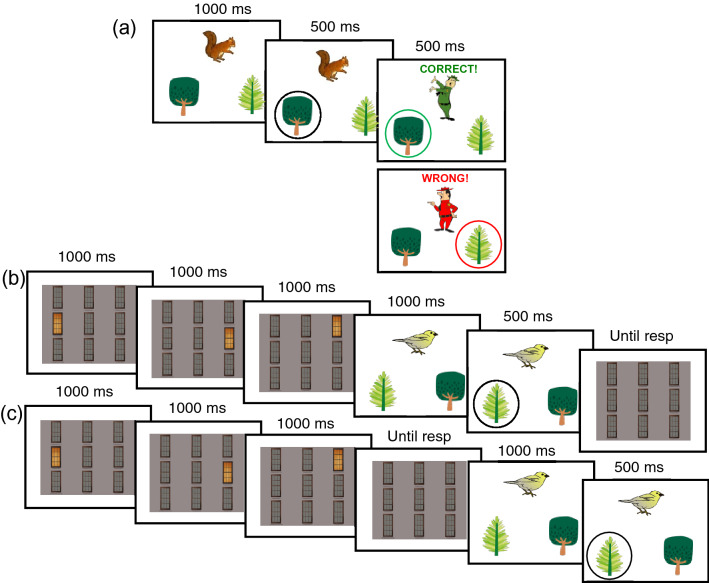
Task design. **a** Successive displays of a typical trial in the Acquired Equivalence Task. The Transfer Testing phase was identical except that no corrective feedback (third frame) was given. **b** and **c** An example trial of the Load and No-Load conditions, respectively. The first three frames for each condition present the study sequence. For **b** (Load condition, Transfer Testing phase) the last frame shows the test display for the WM component of the trial being presented after the Acquired Equivalence Task display, requiring the participant to carry the WM load throughout the trial. For **c** (No-Load condition, Transfer Testing phase) the WM test display is presented before the trial, allowing the WM cache to be unloaded prior to the Acquired Equivalence Task display

For each version (animal, fruit) of the Acquired Equivalence Task, four unique cartoons (2.5° wide, 3.2° high) served as antecedent stimuli (animals: owl, bird, butterfly, and squirrel; fruit: pear, apple, kiwi, and orange) and four cartoons served as unique consequent stimuli (animal version: trees with different shapes and different shades of green; fruit version: grapes with unique bunch shapes and leaves). For each trial (see Fig. 1a), the choice display comprised one antecedent item presented in the center of the upper part of the screen and two different consequent items appearing in the lower half; the background field was always white. Center-to-center horizontal distance between consequent items was 3.8°. The feedback display comprised a cartoon (4.3° wide, 4.8° high) of a happy or angry park ranger (animal version) or a happy or angry bear (fruit version) framed by a green or red circle along with the phrase ‘Correct!’ or “Wrong!” in green or red Arial font (each word: 4.8° wide, 1.4° high) for correct or incorrect responses, respectively. The feedback display appeared in the top half of the screen centered in the same location as previously occupied by an antecedent item.

The WM component during the Transfer Testing phase presented three successive study displays and one test display (see Fig. 1b, c). These displays comprised a gray square with nine gray windows (each 1.9° wide; 3.8° high) arranged in a 3 X 3 matrix. Center-to-center horizontal distance between windows was 3.8° and vertical distance between windows was 0.5°. In study displays, a single window at a time appeared orange. The test display had no orange windows.

### Procedure

On each trial on the Acquired Equivalence Task, one antecedent (animal/fruit) was presented in the upper half of the screen and two consequent items (trees/grapes) were displayed in the lower half. This choice display appeared for 1000 ms, as shown in Fig. 1a. The task was to select a single consequent using the ‘left’ or ‘right’ key on a keyboard. The primary goal of the task was to learn, by trial-and-error, the correct relationship between an antecedent and a consequent. Accuracy was emphasized. The selected item was circled for 500 ms. In all training phases, response feedback was then provided for 500 ms but no feedback was given in the final Transfer Testing phase. At the start of the session, the participant was informed that for each correct answer points would be awarded that could be exchanged for cash (maximum of £3 per task) at the end of the study. A practice block of the Shaping phase with a different set of animal or fruit stimuli, depending on the version of the main task being used, was provided. The practice block terminated after four consecutive correct responses were made.

Participants completed three seamless training phases with no breaks between them: (1) a Shaping phase, (2) an Equivalence Learning phase, and (3) a New Consequent phase. The onset of a new training phase was not signaled to the participant. The three training phases were followed by a Transfer Testing phase. The participants were informed that no corrective feedback was given in this phase and that the phase consisted of 48 trials. The proportion of correct trials for each training phase and for each of two critical trial types (learned and transfer trials) in the Transfer Testing phase was recorded for each participant. Within each phase, the correct consequent was equally likely to be on the left or right; correct consequent location was fully crossed with antecedent item; and all allowable antecedent-consequent combinations were equally likely to occur within a phase and were presented in a pseudorandom order.

In the Shaping phase, there were two possible antecedents, A1 or B1 (e.g., squirrel or bird), and two possible consequents, X1 and Y1 (e.g., tree 1 and tree 2). Each antecedent had only one correct consequent, i.e., A1–X1; B1–Y1. In the Equivalence Learning phase, the possible antecedent set was expanded by adding A2 and B2 (e.g., owl and butterfly), but the consequent set remained limited to X1 and Y1 (tree 1 and tree 2); now both A1 and A2 (squirrel and owl) required X1 (tree 1) as the correct choice and B1 and B2 (e.g., bird and butterfly) required Y1 (tree 2) as the correct choice. For Shaping, the criterion to progress to the next phase was seven correct responses in a row or completing a fixed number of 32 trials. For Equivalence Learning, this criterion was three correct responses in a row or completing a fixed number of 64 trials. In the third phase, the New Consequent phase, two new consequent items (X2, Y2) (e.g., tree 3 and tree 4) were introduced but the possible combinations of antecedent and consequents were constrained. Although A1 (squirrel) was presented with X1 or X2 (tree 1 or tree 3) as a correct choice and B1 (bird) was presented with Y1 or Y2 (tree 2 or tree 4) as a correct choice, A2 (owl) with X2 (tree 3) or B2 (butterfly) with Y2 (tree 4) were never presented. Here, and in the final Transfer Testing phase, no trial required a choice between X1 and X2 (tree 1 and tree 3) or between Y1 and Y2 (tree 2 and tree 4). The criteria to finish the final training phase was 11 correct trials in a row or completion of 96 trials.

The Transfer Testing phase (conducted without feedback) presented all combinations shown in the New Consequent phase as well the previously omitted combinations, specifically A2 (owl) with X2 (tree 3) as correct choice and B2 (butterfly) with Y2 (tree 4) as correct choice. The latter trial types tested transfer learning (12 trials), whereas all other trial types tested association learning (36 trials). Trials in the Transfer Testing phase were combined with a visual spatial WM task. For the latter component, three WM study displays (1000 ms each with no interstimulus interval) were presented at the start of each trial. The participant was instructed to remember the sequence of windows being lit. In the No-Load condition, the WM test array was presented immediately after the last study display and prior to the Transfer Test trial choice display. In the Load condition, the test display was presented *after* the Transfer Test trial. In both cases, the participants reported which windows were illuminated in the correct order by using the computer’s mouse. Participants were asked to report the temporal order and locations. Both the WM component and the Transfer Test had to be correct in order to receive points that were then converted into monetary value at the end of the experiment.

### Statistical analysis

All data from participants who did not reach the learning criterion in the New Consequents phase were excluded (three failed in both conditions; an additional two failed in the Load condition only). However, including all participants (*N* = 27) produced results that were not substantively different (see Supplementary Materials Table S1 and S2). For the remaining participants, data from trials of the Transfer Testing phase were discarded if only one item on the WM test was correctly reported (regardless of WM condition).

Trials to criterion in each training phase and percentage correct for learned trials and transfer trials in the Transfer Testing phase data were analyzed using mixed-effect models. An intercept only model was predicted with a random intercept for each participant. WM condition and Trial Type were dummy coded and added as fixed effect factors (the No-Load condition and learned trials served as reference, respectively). The *lmer* function of the *lme4* R package (Bates et al., 2015) was used to estimate the models. Bootstrapped confidence intervals were obtained with 500 iterations. WM condition order was added to the model of the Transfer Testing phase and was found to have no statistically significant effect (*b* = 0.078, *p* = 0.233) and was therefore not included in the final models. In addition to traditional null hypothesis significance testing, Bayes factors were calculated using Bayesian ANOVAs with subject ID as random factor using default prior probabilities in JASP (version 0.16.1) (JASP Team, 2020). To assess interaction terms, a null model was created with the main effects (WM condition, Trial Type) and subject ID, and compared against the model including the interaction term (WM condition × Trial Type). To assess the role of baseline spatial WM span, forward spatial WM span and backward WM spatial span were separately added to the mixed-effect models and to the null model with the main effects. The Bayes factor BF_10_ is interpreted as a measure of evidence for H_1_ versus H_0_. See Wagenmakers et al., 2017 for guidelines on the interpretation of Bayes factor.

## Results

### Training phases: trials to criterions

As shown in Table 1, for each successive training phase, participants required more trials to reach the learning criteria in subsequent phases (phase 2: *b* = 2.54, 95% CI [ – 4.64, 10.62], *p* = 0.497; phase 3: *b* = 19.38, 95% CI [12.31, 26.62], *p* = 9.12e07; main effect Phase: BF_10_ = 1.41e + 9).

**Table 1:** Trials needed to complete each training phase

	Training phase		
	Shaping (phase 1)	Equivalence learning (phase 2)	New consequents (phase 3)
No WM Load	12.5 (7.0, 18.0)	15.0 (9.5, 20.6)	31.9 (26.4, 37.4)
WM Load	13.8 (8.0, 19.6)	16.6 (10.8, 22.4)	33.3 (27.5, 39.1)

Estimated mean number of trials (95% CI) needed to reach the pre-set learning criteria for each training phase, separated by working memory (WM) condition

Learning in the training phases was comparable across WM conditions (Load, No-Load) (WM condition: *b* = 1.31, 95% CI [ – 5.59, 9.12], *p* = 0.733; main effect WM condition: BF_10_ = 0.21).

### Transfer Testing phase

#### Working memory component

Participants made significantly more errors on the WM component in the Load condition as compared to the No-Load condition (*b* = 4.37%, 95% CI [0.56, 8.12], *p* = 0.025, BF_10_ = 61.98). However, the percentage of errors on the WM component of the trial did not significantly differ between learned and transfer trials (WM condition [WM Load] × Trial Type [Transfer trial]: *b* = 1.19, 95% CI [ – 4.18, 6.57], *p* = 0.660, BF_10_ = 0.29). In the Load condition, 7.9% of the learned trials (SD = 8.2%) and 8.7% (SD = 9.6%) of the transfer trials contained WM component errors. In the No-Load condition this was 3.5% (SD = 3.1%) of the learned trials and 3.1% (SD = 5.4%) of the transfer trials. We further tested the correlation between WM component change (No-Load minus Load condition WM component errors) and Transfer performance change (No-Load minus Load condition transfer accuracy). This correlation appears to be small and non-significant (*r*_*s*_ = 0.171, *p* = 0.447, BF_10_ = 0.48), which was also reflected by the absence of a significant difference in WM component errors between learned and transfer trials.

#### Transfer testing

**Fig. 2:**
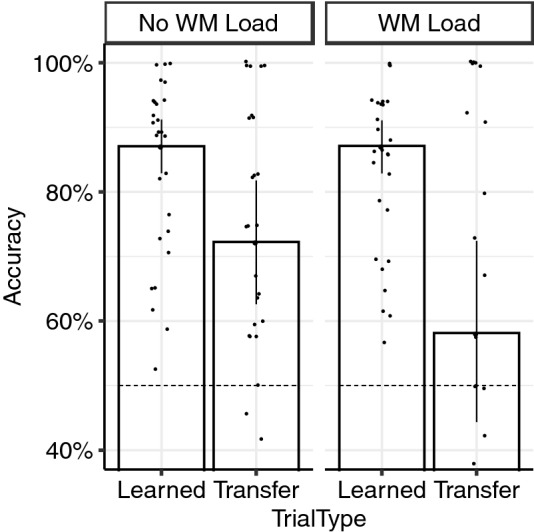
Performance in the Transfer Testing phase. Performance in the Transfer Testing phase for each WM condition (No-Load, Load). Learned trials refer to antecedent-consequent pairs shown in the training phase. Transfer trials are never-trained pairs. Dotted line indicates guess rate of 50% accuracy; error bars represent 95% confidence intervals; dots indicate individual data

As shown in Fig. 2, accuracy in the transfer phase was better for learned (*M* = 87.1%, SE = 4.4%) than for transfer trials (b =  – 14.8, 95% CI [ – 24.25, – 6.46], *p* = 0.002, BF_10_ = 137,639.92), as has been reported previously (e.g., Bódi et al., 2009; Weiler et al., 2008). Importantly, accuracy depended on an interaction between WM condition by Trial Type (b =  – 14.17, 95% CI [ – 28.49, – 1.23], *p* = 0.038, BF_10_ = 1.62). Although performance on learned trials was unaffected by Load condition (Load: *M* = 87.1%, SE = 4.5; No-Load: *M* = 86.1%, SE = 4.6%, *p* = 0.838, BF_10_ = 0.30), group mean accuracy on transfer trials was dramatically lower in the Load condition (*M* = 57.1%, SE = 4.6%) as compared to the No-Load condition (*M* = 72.2%, SE = 4.5%;* p* = 0.002, BF_10_ = 7.64). For the Load condition, performance on transfer trials was not significantly different from chance (*b* = 8.14, 95% CI [ – 2.00, 18.28], *p* = 0.115, BF_10_ = 0.41), whereas it was well above chance for the No-Load condition (*b* = 22.25, 95% CI [13.42, 29.69], *p* = 9.35e-07, BF_10_ = 137.80). While 52.2% (*n* = 12) of the participants in the Load condition performed at or below the 50% chance level for transfer trials, only 16.7% (*n* = 4) of the participants in the No-Load condition performed at or below the 50% chance level. For learned trials, none of the participants in either the Load or No-Load condition performed at or below the 50% chance level.

Adding baseline forward or backward spatial span to the model did not alter the results. The group mean forward spatial WM span was 6.2 (SD = 1.6) and 5.8 (SD = 1.5) for the backward spatial WM span. Exploratory analyses show a correlation between forward spatial WM span and accuracy on transfer versus learned trials (*r*_*s*_ =  – 0.314, *p* = 0.034, BF_10_ = 1.97). The correlation coefficient between backward spatial WM span and accuracy on transfer versus learned trials was *r*_*s*_ =  – 0.099, *p* = 0.514, BF_10_ = 0.24).

## Discussion

We conducted an experiment on young adults to investigate the effect of an experimentally imposed acute WM limitation on transfer of learning. There was clear evidence that when WM was limited (Load condition), transfer learning was impaired. Retention of previously learned associations was not significantly affected by limitations of WM capacity. This result is consistent with the hypothesis that WM is essential for transfer learning, but not for access to prior association learning. The link between WM and processes underpinning transfer learning is further supported by findings that WM performance was worsened in the “Load” condition. By implication, these findings suggest that the process of flexibly applying previously learned associations to new situations may become impaired by reductions in available WM, as occurs when multitasking (Redick, 2016), experiencing stress (Lieberman et al., 2002) or when sleep deprived (Smith et al., 2002).

A possible alternative explanation for these findings is that poor transfer performance was due to a simple increase in task difficulty produced by a secondary task in the Load condition. However, both Load and No-Load conditions required participants to engage in a WM task within each trial of the Transfer testing. The key difference was that in the Load condition, the WM load had to be maintained during the transfer task component of the trial whereas in the No-Load condition, WM could be cleared prior to the transfer trial component. Both conditions required task-switching, and both required keeping in mind a similar set of task instructions. Yet the Load condition produced markedly lower performance on the transfer trial than the No-Load condition. Further supporting the view that this was not due to generalized effects of a dual task condition is the finding that performance of learned trials was unaffected by Load condition.

The current study focused on spatial WM, and it remains to be determined if other types of WM, such as verbal or visual WM, could also contribute to transfer learning. Arguing against this possibility is a study by Weiler et al., (2008) that measured spatial WM and verbal WM in older adults as well as transfer learning, using a similar acquired equivalence paradigm as that used here. They reported that spatial WM was correlated with transfer learning performance, and that spatial WM—but not verbal WM—was significantly lower in the old group compared to the young group. Weiler et al. (2008) further reported a correlation between spatial WM and transfer performance in older adults, but not in younger adults. In the current study, better performance on transfer relative to learned trials was associated with baseline forward spatial WM. Supporting the potential involvement of spatial WM in transfer learning are studies of patients with hippocampal damage, e.g., hippocampal atrophy linked to Alzheimer’s disease or epilepsy patients after surgical resection of the medial temporal lobe. These studies show consistent deficits in transfer learning without major deficits in association learning, leading to the view that the hippocampal area is a critical brain area for transfer learning. Such effects have been reported for even mild cases of hippocampal atrophy with no other cognitive abnormalities (Bódi et al., 2009; Myers et al., 2002, 2003; Weiler et al., 2008). The hippocampal area has likewise been implicated in remembering spatial information, as evidenced by lesion, neuroimaging, neuropsychological, and animal studies (Broadbent et al., 2004; Burgess et al., 2002; Smith & Milner, 1981), suggesting that transfer learning and spatial memory may rely on the same limited-capacity neural areas. However, recent work has shown that the hippocampal area is also central to human verbal WM (Boran et al., 2019), leaving open the possibility that other types of WM may also contribute to transfer learning. Additional studies using verbal or visual WM tasks concurrent with transfer learning tests could be used to investigate this matter.

In sum, using an experimental approach on young adults we showed that transfer of learning depends on access to WM resources and that when these resources are experimentally reduced by imposition of a secondary task, transfer learning suffers.

## Supplementary Information

Below is the link to the electronic supplementary material.

Supplementary file1 (DOCX 39 KB)

## Data Availability

Data will be made publicly available in the Open Science Framework (OSF) repository upon publication and materials are available upon request. The experiment was not pre-registered.
