# Supplementary material for: Working memory load impairs transfer learning in human adults
Source: Psychol Res. 2023 Jan 27;87(7):2138–45. doi: 10.1007/s00426-023-01795-y (PMC10457230; doi:10.1007/s00426-023-01795-y)
Supplement: Supplementary file 1 — Supplementary file1 (DOCX 39 KB) [file 426_2023_1795_MOESM1_ESM.docx]

Working Memory Load Impairs Transfer Learning in Human Adults, Psychological Research, Leonie JT Balter & Jane E Raymond; Department of Clinical Neuroscience, Karolinska Institutet, Stockholm, Sweden, leonie.baltr@ki.se

**SUPPLEMENTARY MATERIALS**

**Table S1.** Accuracy of transfer testing phase

|  |  | Transfer Testing phase | |
| --- | --- | --- | --- |
|  |  | Learned trials | Transfer trials |
| No WM Load | Learners only | 87.1% (78.1, 96.0) | 72.2% (63.3, 81.2) |
|  | All | 83.9% (75.3, 92.4) | 70.7% (62.2, 79.2) |
| WM Load | Learners only | 86.1% (76.8, 95.3) | 57.1% (47.8, 66.3) |
|  | All | 83.5% (74.9, 92.0) | 53.0% (44.5, 61.5) |

*Note.* Results are shown as mean accuracy (95% CI) for learned and transfer trials, separately for the working memory load conditions (No-load, Load) for learners only (those who met the criterion performance) and all participants (learners and non-learners).

**Table S2.** Number of trials needed for each training phase

|  |  | Training phases | | |
| --- | --- | --- | --- | --- |
|  |  | Shaping  (phase 1) | Equivalence Learning  (phase 2) | New Consequents (phase 3) |
| No WM Load | Learners only | 12.5 (7.0, 18.0) | 15.0 (9.5, 20.6) | 31.9 (26.4, 37.4) |
|  | All | 14.1 (6.6, 21.6) | 18.6 (11.1, 26.1) | 39.0 (31.5, 46.5) |
| WM Load | Learners only | 13.8 (8.0, 19.6) | 16.6 (10.8, 22.4) | 33.3 (27.5, 39.1) |
|  | All | 15.6 (8.1, 23.1) | 18.2 (10.7, 25.7) | 44.9 (37.4, 52.4) |

*Note*. Results show number of trials (mean and 95% CI) for each training phase, for the working memory load conditions (No-load, Load) for learners only (those who met criterion performance) and all participants (learners and non-learners). For non-learners, the maximum possible number of trials for each phase was taken as their score (32 trials for the Shaping phase, 64 trials for the Equivalence Learning phase, or 96 trials for the New Consequents phase).
